# Insights into the Mechanisms of Single-Photon and Two-Photon Excited Surface Enhanced Fluorescence by Submicrometer Silver Particles

**DOI:** 10.3390/nano14171451

**Published:** 2024-09-06

**Authors:** Yan Wang, Feng Zhang, Zaifa Du, Xinmin Fan, Xiaodong Huang, Lujun Zhang, Sensen Li, Zhaohong Liu, Chunyan Wang

**Affiliations:** 1 School of Physics and Electronic Information, Weifang University, Weifang 261061, China; wfwy90@wfu.edu.cn (Y.W.); dzfwfu@wfu.edu.cn (Z.D.); xdhuang@wfu.edu.cn (X.H.); wangcy@bnu.edu.cn (C.W.); 2 Weifang Key Laboratory of Laser Technology and Application, Weifang University, Weifang 261061, China; 3 College of Electronic Information and Optical Engineering, Nankai University, Tianjin 300350, China; zf1984266@163.com; 4 Science and Technology on Electro-Optical Information Security Control Laboratory, Tianjin 300308, China; lisensen@cetc.com.cn; 5 Center for Advanced Laser Technology, Hebei University of Technology, Tianjin 300401, China; lzh@hebut.edu.cn

**Keywords:** surface enhanced fluorescence, submicrometer silver particles, single photon excited fluorescence, two photon excited fluorescence

## Abstract

Surface enhanced fluorescence (SEF) based on noble metal nanoparticles is an effective means to achieve high sensitivity in fluorescence detection. Currently, the physical mechanism behind enhanced fluorescence is not fully understood. This paper measures the fluorescence signals of Dihydroporphyrin f methyl ether (CPD4) under both single-photon and two-photon excitation based on submicrometer silver particles with rough morphologies, achieving enhancement factors of 34 and 45 times, respectively. On this basis, by combining the radiative field characteristics produced by the silver particles, a stimulated radiation model of molecules is established to elucidate the changes in the molecular photophysical process when influenced by silver particles. Moreover, the fluorescence lifetime of the molecules was measured, showing that the presence of silver particles induces an increase in the molecular radiative decay rate, causing the fluorescence lifetime to decay from 3.8 ns to 3 ns. The results indicate that the fluorescence enhancement primarily originates from the submicrometer silver particles’ enhancement effect on the excitation light. Additionally, the fluorescence signal emitted by the molecules couples with the silver particles, causing the local surface plasmon resonances generated by the silver particles to also emit light signals of the same frequency. Under the combined effect, the fluorescence of the molecules is significantly enhanced. The findings provide a theoretical foundation for understanding the fluorescence enhancement mechanism of silver particles, adjusting the enhancement effect, and developing enhanced fluorescence detection devices based on submicrometer silver particles, holding significant practical importance.

## 1. Introduction

Optical detection methods based on fluorescence have been widely applied in numerous fields such as biochemical sensing [1,2], dye molecule labeling [3,4], biological detection and imaging [5,6,7]. However, to achieve the highly sensitive detection of fluorescence, samples are generally required to have a large optical absorption cross-section, high quantum efficiency, and good stability [8,9]. In reality, only a few fluorescent materials meet these conditions, and the luminescence efficiency of many materials is very low, leading to their ineffective detection. Therefore, there is a demand for a means to efficiently enhance the luminescence intensity of fluorescent materials.

Among various strategies, the surface plasmon enhanced effect of noble metals is a very important and effective approach [10,11,12]. Noble metal nanoparticles such as gold, silver, and copper possess unique dielectric function properties. Under specific wavelengths of light excitation, free electrons on the surface collectively oscillate. When the oscillation frequency matches the intrinsic oscillation of free electrons within the metal particles, a strong localized field forms on the surface of the noble metal nanoparticles, known as localized surface plasmon resonance (LSPR) [12]. The LSPR effect has given rise to surface enhanced Raman scattering (SERS) [13,14,15,16] and surface enhanced fluorescence (SEF) [17,18,19,20]. Under the action of localized surface fields, fluorescence, Raman scattering, and other effects of molecules surrounding the nanoparticles are significantly enhanced. Historically, due to the inherently weak Raman scattering signals and the more pronounced enhancement effect of SERS, theoretical and application research on SERS has been very extensive, while studies on SEF have been relatively scarce [21].

The radiative process of fluorescent molecules in the excited state is closely related to the electromagnetic field in their surrounding environment. Drexhage was the first to investigate the luminescence behavior of fluorescent molecules near the surface of silver films, finding that their fluorescence lifetime oscillates with the distance between the molecules and the interface, experimentally confirming that the local electric field on the surface of noble metals can cause changes in molecular fluorescence [22]. Professor Lakowicz’s group at the University of Maryland, USA [23,24,25,26,27] conducted extensive research on the SEF phenomenon from both theoretical and experimental perspectives. By studying the effect of metal nanostructures on enhancing fluorescence experimentally and interpreting the phenomena theoretically, they laid the foundation for research into the enhancement of fluorescence by metal nanostructures. With continuous advancements in metal nanotechnology and deeper research into the theory of surface plasmons, a variety of metal nanostructures have emerged, prompting increasing research into SEF [19,28,29,30,31].

Studies show that the local electromagnetic environment around metal nanoparticles affects the spontaneous radiation process of molecules [32]. Under the influence of an electromagnetic field, the absorption of light by fluorescent molecules is significantly enhanced, and the intensity of the fluorescence radiation signal is greatly increased. Additionally, the presence of metal particles may cause an accelerated radiation rate of fluorescent molecules and exhibit directional radiation characteristics [33,34]. In summary, it is widely recognized that the interaction between metal nanoparticles and quantum systems (such as fluorescent molecules, atoms, quantum dots) is the main factor causing changes in the optical properties of fluorescent molecules. Researchers have conducted numerous studies on the mechanism by which metal particles affect the photophysical process of fluorescent molecules, but a unified and clear explanation has not yet been provided. Recent studies have found that since molecular fluorescence involves physical processes such as light absorption and emission, the generation of SEF cannot be explained by a single physical mechanism, but is more likely a combined effect [30,31]. For example, in 2021, Wenchao Zhao demonstrated that metal films enhance the absorption of incident photons at the excitation wavelength of quantum dots, while surface plasmons increase the radiation rate at the emission wavelength of quantum dots [30]. This combined effect significantly enhances the fluorescence of quantum dots. Due to the unclear enhancement mechanism, there are many problems in the study of surface-enhanced fluorescence, the most typical being that the enhancement factors calculated theoretically are much higher than the experimental observations. Therefore, studying the interaction mechanism between metal nanostructures and fluorescent molecules based on the characteristics of surface plasmons, at the level of basic scientific problems, is very meaningful for subsequently optimizing nanostructures, accurately and effectively controlling the enhancement effect, and thus achieving highly sensitive detection with fluorescence technology [35,36].

In this study, the dihydrochlorin f ether (2-devinyl-2-(1-methoxyl-ethyl) chlorin f, CPD4) was used as the research subject to analyze the mechanism of surface-enhanced fluorescence on the rough surface of submicrometer metal particles. We measured the surface-enhanced fluorescence spectra produced by submicrometer silver particles under single-photon and two-photon laser conditions. By adjusting the intensity of the excitation light, we obtained the curve of surface-enhanced fluorescence as a function of excitation light intensity and analyzed the physical mechanisms of surface-enhanced fluorescence. Concurrently, focusing on the interaction between the localized field generated by metal particles and the fluorescent molecules, we established a model for the stimulated radiation of molecules. Theoretical derivations of the changes and patterns in the molecular radiation decay rate under field action were conducted, and experimental validation was performed by measuring the fluorescence lifetime curves of the molecules. The results indicate that the enhancement effect of submicrometer silver particles on the excitation light plays a primary role in enhancing fluorescence. At the same time, the coupled radiation between molecular fluorescence and metal particles, as well as the increased radiation decay rate caused by stimulated radiation of the molecules, also contribute to the enhancement of fluorescence.

## 2. Materials and Methods

### 2.1. Synthesis of Submicrometer Silver Particles

Utilizing a recognized chemical reduction method [37], submicrometer silver particles were produced from silver nitrate. Initially, we combined 0.6 mL of 1 M AgNO_3_ with 6 mL of 100 mM polyvinylpyrrolidone in 30 mL of distilled water. With persistent stirring at ambient temperature, 0.6 mL of 1 M ascorbic acid was promptly added. Once the solution turned dark gray, indicating the end of the reaction (approximately after 8 min), the mixture was centrifuged at 4000 rpm for 15 min to obtain the particles. To rid the synthesized particles of solvent remnants, they were washed with pure water and centrifuged thrice, ensuring almost pure silver particles.

### 2.2. Deposition of Submicrometer Silver Particles

Adopting the self-assembly technique [38], these particles were then deposited onto a photovoltaic substrate. The quartz substrate underwent an extensive cleaning process: first, with detergent, followed by multiple washes with distilled water, and finally with ethanol. Each washing step involved ultrasonic cleaning for 10 min. Post-cleaning, the substrate was dried using nitrogen gas.

A 1% volume fraction polymer solution was created using 3-Aminopropyl trimethoxysilane and water. The cleaned substrate was soaked in this solution, followed by washing with methanol and copious amounts of ultrapure water, and then dried with nitrogen gas.

For the final sedimentation step, the synthesized silver particles were diluted in water and mixed well. The substrate was then submerged in this particle solution inside a nitrogen-filled sealed glove box for a minimum of 2 h. Post-immersion, excess water was removed, and the substrate was air-dried using nitrogen gas.

### 2.3. Optical Modeling

**Figure 1:**
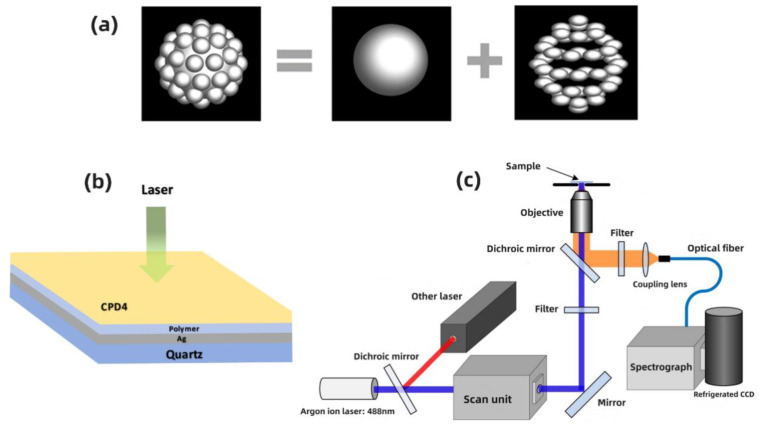
(**a**) Illustrative diagrams depicting flower-like silver particles. (**b**) Schematic of sample structure. (**c**) Schematic of the detection system.

Building on prior investigations, the silver particles are bifurcated into two distinct segments: the core particle, which is about 400 nm in dimension, and the surface outgrowths, each measuring roughly 100 nm. This configuration ensures the aggregate dimension of the particle remains at 500 nm [39], as delineated in Figure 1a.

The localized surface fields of the silver particles were ascertained using the three-dimensional finite difference time domain (FDTD) method. The dielectric constants for silver were sourced from Palik’s findings [39,40]. A Perfectly Matched Layer (PML) was utilized for boundary conditions. The input light was set to be incident in the positive z-direction and was polarized along the x-axis.

### 2.4. Preparation of Samples

First, CPD4 was dissolved using a minuscule amount of 0.1 N NaOH. The concentration of CPD4 was then adjusted to 5 × 10^−6^ mol/L, appropriate for experimental measurements, using saline solution, and its pH value was fine-tuned to 7.0. Employing a pipette, 5 μL of the CPD4 solution was placed on a clean cover glass (18 mm × 18 mm). By carefully moving the substrate sprinkled with sub-micron silver particles towards the cover glass, capillary and adhesive forces ensured that the CPD4 solution was uniformly spread between the overlapping region of the cover glass and the enhanced substrate. This resulted in the cover glass becoming tightly adhered to the functional component.

In the preparation process, polyvinylpyrrolidone was added, forming a polymer layer between the metal particles and the fluorescent molecules, as shown in Figure 1b. This ensures a suitable distance is maintained between the fluorescent molecules and the metal particles, effectively suppressing energy transfer between them and preventing fluorescence quenching.

### 2.5. Instrumentation

The concentration of the CPD4 solution was set to 5 × 10^−6^ mol/L. As shown in Figure 1c, for the experiments, single-photon excited fluorescence (SPEF) and two photon excited fluorescence (TPEF) of the samples were excited using a 488 nm argon-ion laser and a titanium-sapphire femtosecond laser (Coherent Mira 900, Santa Clara, CA, USA) with a central wavelength of 800 nm as the excitation light sources, respectively. The switching between different light sources was achieved through a dichroic mirror in a confocal scanning unit. A 40× objective lens was used to excite and collect the molecular fluorescence signals, which were then spectrally dispersed by a grating spectrometer (Spectropro 300i, Acton, MT, USA) and recorded by a liquid nitrogen-cooled CCD (PI, San Francisco, CA, USA). When measuring the pure CPD4 fluorescence signal and the surface-enhanced fluorescence signal under the action of metal particles, the integration times used by the CCD were 500 ms and 50 ms, respectively. For the measurement of TPEF, the femtosecond laser had a pulse width of 120 fs, a repetition rate of 76 MHz, and an output average power of 630 mW.

Fluorescence lifetime measurements were performed on the DeltaFlex ultrafast fluorescence lifetime detection system from HORIBA. A pulsed laser with a central wavelength of 400 nm and a repetition rate of 1 kHz was chosen as the excitation source. Scanning electron microscopy (SEM) images of the specimens were acquired with a JEOL JSM-6700f scanning electron microscope at 3.0 kV.

## 3. Results and Discussion

### 3.1. Enhancement Effect of Submicrometer Silver Particles under Both Single-Photon and Two-Photon Excitation

**Figure 2:**
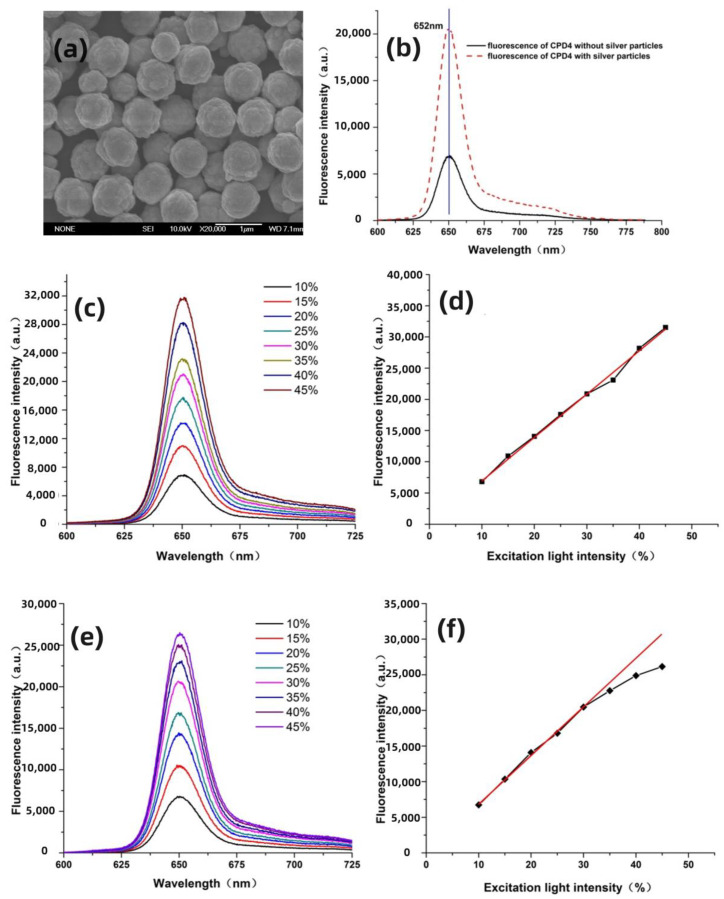
(**a**) The SEM image of the submicrometer silver particles (the scale bar = 1 μm). (**b**) Experimentally measured CPD4 SPEF spectrum with and without silver particles. (**c**) SPEF spectrum of CPD4. (**d**) Variation curve of SPEF spectrum peak intensity at 652 nm. (**e**) The enhanced SPEF of spectrum CPD4 with varying excitation light intensity. (**f**) Variation curve of the enhanced SPEF spectrum peak intensity at 652 nm.

The SEM of submicrometer silver particles was first measured, as shown in Figure 2a. Our previous work conducted detailed experimental and theoretical research on the optical properties of submicron silver particles [40,41]. The results show that they have good absorption characteristics in the ultraviolet–visible wavelength range and can effectively enhance the absorption [42], fluorescence and Raman signals of samples. Also, the rough surface morphology structure affects the distribution of the localized surface field, and submicrometer silver particles possess a strong localized field distribution and a wide spectral range of plasmon resonance frequencies. This allows for a better match with a broader range of molecular fluorescence spectra, and is suitable for multiple excitation wavelengths.

CPD4 is a second-generation porphyrin photosensitizer. Due to its high singlet oxygen yield and significant killing effect on tumor cells, along with its well-defined structure and active ingredients, it has good clinical application prospects. Figure 2b shows the SPEF spectrum of CPD4 and the enhanced SPEF measured on the substrate of submicrometer silver particles. It is observed that under the action of silver particles, the molecular fluorescence is significantly enhanced, while the shape and peak position of the fluorescence spectrum remain unchanged. By calculating the measured fluorescence intensity and the CCD integration time, it was found that the fluorescence of CPD4 was enhanced by about 34 times. The localized surface field, induced by the excitation light and molecular fluorescence, plays a decisive role in fluorescence enhancement. In the experiments, by adjusting the excitation light intensity with a tunable attenuator, the variation of molecular fluorescence signal with excitation light intensity was measured to study the role of these two factors in inducing localized surface field in surface-enhanced fluorescence. Therefore, the fluorescence spectra of CPD4 were measured when the excitation light intensity varied from 10% to 45%, as shown in Figure 2c.

The results in the figure indicate that under single-photon excitation conditions, the intensity of molecular fluorescence is directly proportional to the intensity of the excitation light, i.e., the molecular fluorescence linearly increases with the increase in excitation light intensity. By changing the intensity of the excitation light, the curve of the fluorescence intensity of CPD4 with the change in excitation light intensity was obtained, and a linear fit of the curve was performed, as shown in Figure 2d. The results show that in the absence of silver particles, the fluorescence intensity of CPD4 changes linearly with the excitation light intensity, which is consistent with the principle of fluorescence production. When silver particles are present, the curve of fluorescence intensity changes significantly, as shown in Figure 2e,f. In the region of lower excitation light energy (light intensity below 30% of laser energy), the SEF of CPD4 still shows a linear increase, consistent with the change in fluorescence signal. When the excitation light energy is further increased, the increase in SEF intensity of CPD4 becomes slow, showing obvious saturation characteristics.

When the energy of the excitation light is weak, metal particles can effectively enhance the intensity of the excitation light, thus enabling fluorescent molecules to absorb more strongly, leading to a noticeable increase in fluorescent radiation, which increases linearly with the intensity in the excitation light. In this case, the enhancement of the excitation light by metal particles plays a primary role. However, as the excitation light further strengthens, due to the limited number of fluorescent molecules involved in excitation, they cannot effectively absorb and utilize the localized field near the metal particles, resulting in a saturation absorption effect. Therefore, if the primary role of metal particles is to enhance fluorescent radiation, then the intensity of the excitation light at this point should not induce saturation absorption in the molecules, and thus the fluorescence intensity curve would continue to rise linearly without exhibiting saturation. From this, it can be inferred that in this experiment, when the energy of the excitation light is weak, the enhancement of CPD4 fluorescence mainly originates from the enhancement effect of the submicrometer silver particles on the excitation light.

According to the saturation absorption effect of molecules, when fluorescent molecules undergo saturation absorption, their fluorescence signal will no longer continue to increase but will remain constant. However, in our experiments, the measured SEF signal still showed a slight continuous increase. Previous research has indicated that fluorescent molecules might transfer some of their energy to metal nanoparticles, causing the metal particles to emit light of the same frequency, thereby enhancing the detected fluorescence signal. This process will cause the fluorescence lifetime of the molecules to decay and enhance the fluorescence signal. Therefore, it can be speculated that the fluorescence produced by the molecules couples with the localized field generated by the metal particles, causing the metal particles to radiate a field with the same frequency as the fluorescence signal. Since the energy for the metal particles to generate surface plasmons comes from the excitation light, the enhanced fluorescence signal can be increased over a larger range, but the magnitude of increase is smaller than the increment produced when the light intensity is weaker.

**Figure 3:**
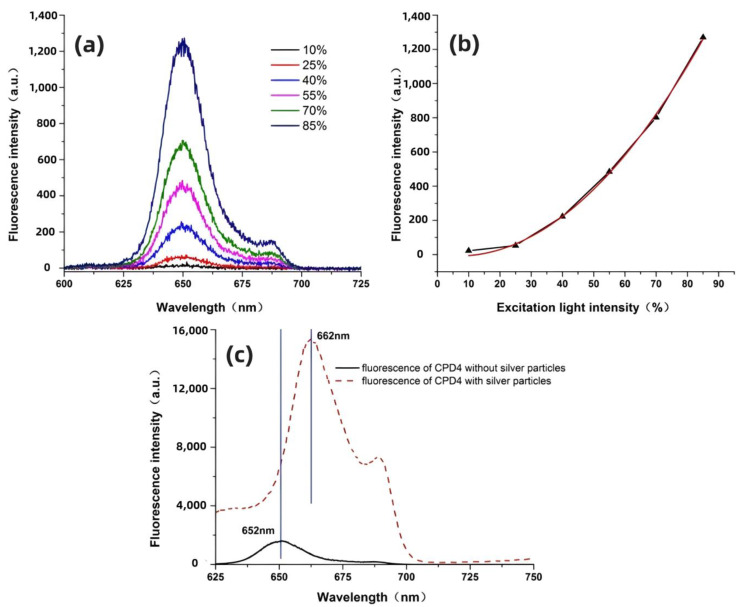
(**a**) The TPEF spectrum of CPD4 with varying excitation light intensities. (**b**) The curve of change in fluorescence peak intensity at 652 nm. (**c**) The TPEF spectrum of CPD4 alongside the enhanced TPEF spectrum.

Currently, the surface enhanced nonlinear effects of noble metals are also a major focus of many researchers. Among these, the TPEF is renowned for its high spatial resolution [39]. Compared to the signal intensity produced by traditional SPEF, the emission intensity of TPEF is significantly lower, which greatly reduces its application in high-sensitivity detection [31]. The emergence of surface-enhanced spectroscopy has paved the way for the development of TPEF. Figure 3a displays the TPEF spectrum of CPD4. The results indicate that the TPEF spectrum of CPD4 is essentially consistent with its SPEF spectrum, but a weak side peak appears near 685 nm. After repeated experiments, the side peak was confirmed to exist, though its cause remains unclear. The quadratic curve fitting results shown in Figure 3b demonstrate that the peak intensity of TPEF is directly proportional to the square of the excitation light intensity. On this basis, we measured the surface-enhanced TPEF spectrum of CPD4 on a silver particle substrate, as shown in Figure 3c. Under the influence of silver particles, the TPEF signal of CPD4 was significantly enhanced, with an enhancement factor of up to 45 times. Additionally, due to the presence of silver particles, the fluorescence peak of CPD4 shifted by about 9 nm. Similar research results have been reported in the literature, but without a clear analytical explanation [43,44].

In the case of single photon excitation, the absence of red-shift in the fluorescence peak suggests that silver particles may not be the primary cause of changes in the molecular fluorescence radiation process. The field generated by different excitation lights might lead to changes in the molecular energy level structure. For femtosecond-scale pulse lasers, a single pulse possesses extremely high energy and peak power. Taking the 10% light intensity excitation in the experiment as an example, after system losses and focusing through a microscope objective, the laser spot size is about 1 μm^2^, and the peak power at the focus is approximately 1 × 10^9^ W/cm^2^. Under the enhancement effect of metal particles, a stronger localized field distribution will form around the molecules. Due to the intense electromagnetic field, the inherent dipole moment of fluorescent molecules near the metal particles may change, leading to a shift in their potential energy curves and, consequently, a red shift in the fluorescence radiation.

### 3.2. Theoretical Analysis and Molecular Excited State Dynamics Process Induced by Localized Surface Field

**Figure 4:**
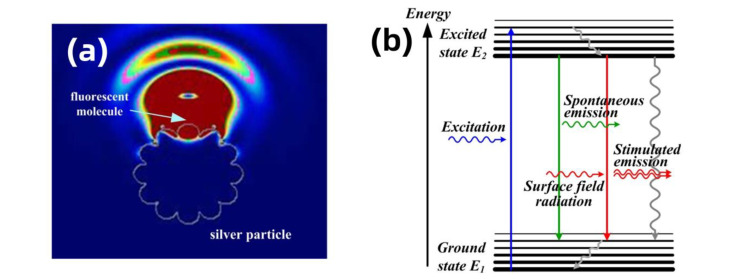

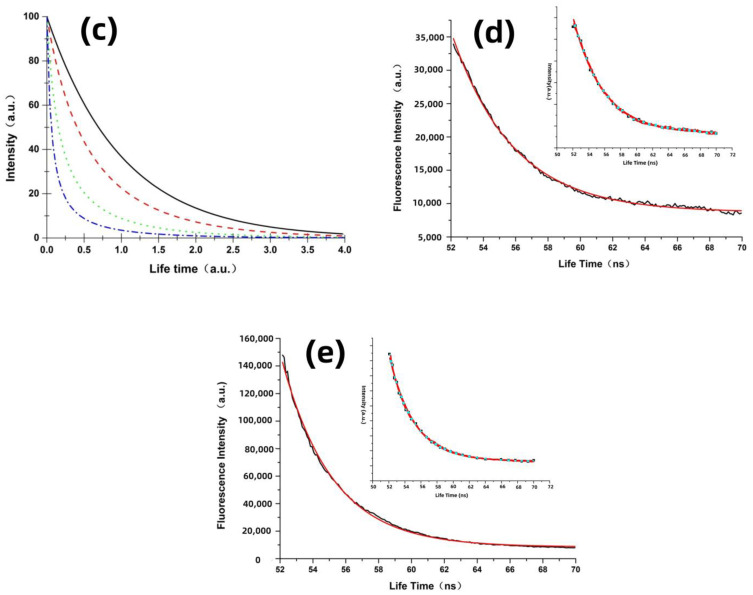
(**a**) Surface local field distribution induced by molecular fluorescence in submicrometer silver particles. (**b**) Photophysical process of excited fluorescent molecules under the influence of silver particles. The blue solid line represents the stimulated absorption process, the green solid line represents the spontaneous radiation process, the red solid line represents the stimulated radiation process, and the gray curve represents the non-radiative transition. (**c**) Simulation showing that as the value of k increases, the molecular fluorescence lifetime changes. (**d**) Change curve of spontaneous fluorescence lifetime of CPD_4_ molecule. The embedded images display both the scatter and line graphs of the experimental data. (**e**) Change curve of fluorescence lifetime of CPD_4_ molecule under the action of silver particles. The embedded images display both the scatter and line graphs of the experimental data.

Fluorescent molecules might transfer part of their energy to metal particles, causing the metal particles to emit light of the same frequency, thus enhancing the detected fluorescence signal. This process will lead to a decay in the molecular fluorescence lifetime and enhance the fluorescence signal. The following sections explore this physical process from both theoretical and experimental perspectives. The fluorescence radiation of molecules can be approximated as dipole oscillation radiation. Using the dipole model used to represent fluorescent molecules, we utilized FDTD software to simulate the localized field distribution on the metal particle surface caused by molecular fluorescence when the fluorescent molecules are located near the metal particle surface, as shown in Figure 4a. From the figure, we can see that a pronounced field oscillation and outward radiation form in a tiny region of the submicrometer silver particle surface adjacent to the fluorescent molecule. This oscillation frequency matches the molecule’s fluorescence radiation. It is known that excited-state molecules will undergo stimulated radiation under the influence of light at the same frequency as their radiation. As the fluorescent molecules are within the local field produced by submicrometer silver particles, this local field might induce stimulated radiation in fluorescent molecules, leading to fluorescence lifetime decay.

Based on the stimulated radiation model of molecules, we studied the radiation transition process of molecules. As one of the most fundamental equations in quantum mechanics, Fermi’s golden rule provides the probability of a molecule transitioning from an excited state back to the ground state [45],

(1) $$w_{if}\left(t\right)=\frac{{\left|\mu_{if}\right|}^{2}\rho \left(\upsilon \right)}{6\varepsilon_{0}\hbar^{2}}$$

where $\mu_{if}$ is the matrix element determined by the molecular wave function, representing the transition term between the initial state (*i*) and the final state (*f*). $\rho \left(\upsilon \right)$ represents the energy density of the incident field at the transition frequency. While Fermi’s rule provides the transition probability of molecules, the photo physics of this process requires analysis using the molecular rate equation. Therefore, Einstein’s radiation model is further analyzed. Figure 4b illustrates three transition processes in a two-level system. The Einstein coefficients for the three transition processes of stimulated absorption, stimulated radiation, and spontaneous radiation are represented by *B_12_*, *B_21_*, and *A_21_*, respectively. In the Einstein model, the probability of the stimulated transition of the molecule is represented as

(2) $$w_{if}=B_{if}\rho \left(\upsilon \right)$$

Comparing the transition probability in Fermi’s golden rule with Einstein’s model, it can be observed that when

(3) $$B_{if}={\left|\mu_{if}\right|}^{2}/6\varepsilon_{0}\hbar^{2}$$

Fermi’s golden rule simplifies into Einstein’s radiation model. Building on Einstein’s radiation model, we analyze the fluorescence lifetime of molecules. Assuming the total number of molecules is *n*, based on the processes represented in Figure 4b, we can write the rate equation for the number of molecules at each energy level as a function of time, as follows:

(4) $$\begin{array}{l} \frac{dn_{2}}{dt}=n_{1}w_{12}-n_{2}\left(A_{21}+w_{21}\right)\;(a)\\ n_{1}+n_{2}=n\;(b) \end{array}$$

where n_1_ and n_2_ are the numbers of molecules in the ground and excited states, respectively. When the surrounding environment is free space, the fluorescent molecule will not produce stimulated radiation, that is, $w_{21}=0$. After being acted upon by a pulse laser, the molecule has no absorption, that is, $w_{12}=0$. At this time, only the spontaneous radiation process of the excited state molecule needs to be considered. Therefore, under this transient non-equilibrium state, the rate equation for the excited state molecule given by Equation (4a) is

(5) $$\frac{dn_{2}}{dt}=-An_{2}$$

Solving this, we get

(6) $$n_{2}\left(t\right)=n_{20}e^{-At}$$

where $n_{20}$ is the number of molecules in the excited state at the initial moment. Since the fluorescence emitted by the molecule is proportional to the number of excited-state molecules undergoing radiative transition, that is, $w_{12}=0$, the fluorescence decay curve is

(7) $$I\left(t\right)=I_{0}e^{-At}$$

From Equation (7), the spontaneous fluorescence lifetime is

(8) τ0=1A

When the molecule is near the sub-micron silver particle, the fluorescence emitted by the molecule is localized on the surface of the metal particle and induces the metal particle to produce radiation of the same frequency. Under the local field of the same frequency generated by the molecule on the metal surface, the molecule will undergo stimulated radiative transition. Therefore, considering the effect of stimulated radiation, the change in the number of excited-state molecules is revised to

(9) $$\frac{dn_{2}}{dt}=-\left(A+w_{21}\right)n_{2}=-\left(A+B\rho \right)n_{2}$$

Here, the light field density is induced by the fluorescence emitted by the molecule on the surface of the metal particle. For simplification, the radiative field density produced by the metal particle is proportional to the fluorescence emitted by the molecule, that is, ρ=kI. Here, k is a proportionality constant, proportional to the coupling interaction between the metal particle and the fluorescent molecule (including the enhancement effect of the metal particle on fluorescence) and the percentage of fluorescence acting on the metal particle in the total fluorescence energy. So Equation (9) is

(10) $$\frac{dn_{2}}{dt}=-\left(A+BkI\right)n_{2}$$

This ignores the effect of the number of excited-state molecules on fluorescence changes. Therefore, under the induction of the local field produced by the metal particle, the fluorescence decay curve becomes

(11) $$I\left(t\right)=\frac{AI_{0}}{\left(BkI_{0}+A\right)e^{At}-BkI_{0}}$$

The fluorescence lifetime of the molecule under metal particle enhancement is calculated as

(12) $$\tau =\frac{1}{A}\ln\frac{Ae+BkI_{0}}{A+BkI_{0}}$$

From the expression of fluorescence lifetime under metal particle enhancement, it can be seen that $1<\frac{Ae+BkI_{0}}{A+BkI_{0}}<e$, indicating $\tau <\tau_{0}$, and suggesting that the fluorescence lifetime has decayed.

Equation (11) indicates that the decay of the molecular fluorescence lifetime is directly related to the stimulated radiation Einstein coefficient of the molecule and the local field enhancement factor of the metal particles. As the Einstein coefficient is only related to the properties of the molecule itself, we use k as a variable and simulate the change in molecular fluorescence lifetime based on Equations (7) and (11). As shown in Figure 4c, as parameter k increases, the molecular fluorescence lifetime decreases significantly. Thus, the coupling between metal particles and molecules plays a crucial role in reducing the molecular fluorescence lifetime.

From the aforementioned theoretical analysis, it is evident that the fluorescence emitted by the fluorescent molecule causes the formation of a local surface field oscillation in the surface area of the metal particle, which radiates light of the same frequency. The field produced by the metal particle, in turn, acts on the molecule, causing the excited-state fluorescent molecule to produce stimulated radiation, thereby leading to an increase in the radiative transition rate of the excited-state fluorescent molecule. Compared to the spontaneous radiation lifetime produced by the molecule, the surface field of the metal particle causes a decay in the lifetime of the fluorescent molecule. At the same time, the increased radiation rate results in an increased number of molecules returning to the ground state per unit time, further enhancing the molecule’s light absorption. The stronger the coupling between the metal particle and the fluorescent molecule, the shorter the lifetime of the fluorescent molecule, and the stronger the absorption, i.e., the increase in the molecular radiation rate. Thus, we believe that the presence of metal particles causes significant changes in the photophysical properties of the molecule.

To further verify the changes in molecular fluorescence lifetime under the action of metal particles, we measured the fluorescence lifetime curves of CPD4 with and without the action of metal particles, as shown in Figure 4d,e. The embedded images display both the scatter and line graphs of the experimental data. It is visually apparent that the line graph fits the original experimental data very well, with minimal error, allowing for the analysis of fluorescence lifetime based on the line graph. 

The fluorescence lifetime decay curve is approximately fitted with the following bio-exponential decay function [46]:

(13) $$I=I_{0}+A_{1}\exp^{\frac{-(t-t_{0})}{\tau_{1}}}+A_{2}\exp^{\frac{-(t-x_{0})}{\tau_{2}}}$$

where $\tau_{1}$ and $\tau_{2}$ are the lifetimes of two different decay channels, representing the spontaneous emission lifetime and the lifetime due to the coupling effect between metal particles and fluorescent molecules, respectively. Additionally, A_1_ and A_2_ represent the amplitude values. The average lifetime can be calculated using the following formula [46,47]:

(14) $$\tau =A_{1}\tau_{1}+A_{2}\tau_{2}$$

Table 1 shows the parameter values obtained by fitting the experimental data basedon Equation (13). The values are substituted into Equation (14); we can then can calculate the average lifetime of the CPD_4_ fluorescent molecule as 3.8 ns, and when metal particles are present, the fluorescence lifetime of CPD_4_ decays to 3 ns. The experimental results are consistent with the theoretical simulation, further proving that the coupling between sub-micron silver particles and molecules causes a significant decay in the molecular fluorescence lifetime.

## 4. Conclusions

In this study, the submicrometer silver particles were utilized to prepare substrates for enhancing both the SPEF and TPEF spectra of CPD4. By adjusting the intensity of the excitation light, we obtained the curve of SEF as a function of excitation light intensity and analyzed the physical mechanisms behind SEF. Furthermore, in consideration of the interaction between the localized surface field generated by metal particles and the fluorescent molecules, a stimulated radiation model of molecules was established. Theoretical derivations of the changes and patterns in the molecular radiation decay rate under field action were conducted, and experimental validation was performed by measuring the fluorescence lifetime curves of the molecules. The following research results were obtained.

1. Under the influence of silver particles, the SPEF signal of molecules was enhanced by 34 times. The intensity of SEF grew linearly with the excitation light, but exhibited saturation characteristics with a slight increase at high excitation power. It can be inferred that the fluorescence enhancement primarily originates from the enhancement effect of silver particles on the excitation light, while the fluorescence signal emitted by the molecules couples with the silver particles, inducing them to emit light of the same frequency, thereby significantly enhancing the fluorescence signal of the tested molecules. Under two photon excitation conditions, the molecular fluorescence signal could be enhanced by 45 times, and the SEF peak exhibited a red shift of 10 nm, speculated to be due to changes in the molecules’ inherent dipole moment caused by the strong light field.

2. Combining the characteristics of the radiation field generated by silver particles, a stimulated radiation model of molecules was established, elucidating the changes in the molecular photophysical process when acted upon by metal particles. The experimental results show that silver particles caused the molecular fluorescence lifetime to decay from 3.8 ns to 3 ns. This indicates that the radiation field induces an increase in the molecular radiation decay rate. The extent of change in the molecular radiation decay rate has a direct relationship with the molecules’ spontaneous fluorescence lifetime and the coupling effect between the metal particles and fluorescent molecules. The greater the coupling effect, the higher the radiation decay rate, and the more significant the lifetime decay observed in fluorescent molecules with longer spontaneous fluorescence lifetimes.

In summary, submicrometer silver particles can significantly enhance molecular fluorescence. The energy of the excitation light plays a primary role in fluorescence enhancement, while the coupled radiation between molecular fluorescence and silver particles, as well as the increased radiation decay rate caused by the stimulated radiation of the molecules, also contribute to fluorescence enhancement. These results provide strong theoretical support for the subsequent regulation of the enhancement effect of submicrometer silver particles.

## Figures and Tables

**Table 1:** Fluorescence lifetime parameter values fitted by the bio-exponential function.

Samples	A_1_	$\tau_{1}$ (ns)	Standard Error	$\tau_{2}$ (ns)	Standard Error	A_2_	τ	R-Square	Confidence Level
CPD_4_	0.53	3.46	0.017	4.19	0.014	0.47	3.8	0.991	95%
CPD_4_ with the silver particles	0.56	3.32	0.019	2.59	0.016	0.44	3	0.993	95%

## Data Availability

The raw data supporting the conclusions of this article will be made available by the authors on request.
